# Supplementary material for: An anti-occlusion optimization algorithm for multiple pedestrian tracking
Source: PLoS One. 2024 Jan 31;19(1):e0291538. doi: 10.1371/journal.pone.0291538 (PMC10830026; doi:10.1371/journal.pone.0291538)
Supplement: Attachment — Submitted filename: a-plos one-Response-to-Reviewers-v2.docx [file pone.0291538.s001.docx]

**Original Manuscript Number:** PONE-D-23-09646

**Original Article Title: “An Anti-Occlusion Optimization Algorithm for Multiple Pedestrian Tracking”**

**To:** Plos One Editor

**Re:** Response to reviewers

Dear Editor and Reviwers,

Thank you for allowing a resubmission for our manuscript, with an opportunity to address the reviewers’ comments.

We have attached the following documents: (a) our point-by-point response to the reviewers’ comments (response to reviewers), (b) an updated manuscript with yellow highlighting indicating changes (*Supplementary Material for Review*), and (c) a clean updated manuscript without highlights (*Main Manuscript).*

In order to comply with the copyright requirements of the journal, we have modified the portrait display in the image.

Wish you happy everyday!

Best regards,

Lijuan Zhang, Gongcheng Ding, Guanhang Li, Yutong Jiang, Zhiyi Li, Dongming Li

We would like to express our gratitude for granting us the opportunity to resubmit our manuscript and address the valuable comments provided by the reviewers. We have attached the following documents:

(a) Our point-by-point response to the reviewers' comments (Response to Reviewers).

(b) An updated manuscript with highlighted changes in yellow (Supplementary Material for Review).

(c) A clean updated manuscript without highlights (Main Manuscript).

We wish you a pleasant day ahead!

Best regards

**Reviewer#1, Concern # 1:** Some figures, such as Figs. 1, 2, are unclear. The details of the blocks should be clearly defined and explained directly in the figures even they have been explained in text. Please provide the information as much as possible, then the readers can get the details from each separate figure easily even without the text. What do the circles mean? What do the colors of the three feature boxes mean?.

**Author response:** Thank you very much for your valuable comments!

We modified the model diagram according to your suggestion, and revised the vague module expression. We have corrected the use of circles, and replaced the relevant operation descriptions with MatMul and Concat. In our proposed model, we use cyclic shift to generate negative samples, and we have updated the module in Figure 2. The colors of the three feature boxes are used to express feature information of positive and negative samples to the reviewers and readers and the subsequent operation of splicing on the channel. At the same time, we have drawn a detailed network model diagram. Now you can clearly know the relevant information of each module, and the new model diagram is shown in Figure 2.

**Author action:** We have updated the model diagram in the manuscript, and the updated model diagram is shown in Figure 1.

Fig.1:Feature extraction model diagram.

Fig.2: Cycle-attention module.

**Reviewer#1, Concern # 2:** The characters in Fig. 6 are too small. Please check it and revise it. What does the circle (with x) mean?

**Author response:** Thank you very much for your comments!

We have revised the flowchart based on your comments, replacing vague symbolic representations. We redraw the flow chart, correct the vague symbols and change them to detailed text descriptions，and add more precise descriptions of judgment conditions in our revised manuscript. Thank you very much for the comments.

**Author action:** We have modified the flow chart, and the modified part is shown in Figure 6:

Fig.6: Matching flow chart.

**Reviewer#1, Concern # 3:** Please check the manuscript carefully to remove the typos, improve the language and format.

E.g.

-Wes uses

**Author response:** Thank you very much for the comments.

We have carefully checked the English language and style for our manuscript.

**Author action: Done.** We have revised our manuscript as your comments.

**Reviewer#1, Concern # 4:** The review of the related works and comparison experiments can be more sufficient. Please carefully read, cite and compare (if applicable) the following papers that are SOTA tracking. [-SiamCAM: A real-time Siamese network for object tracking with compensating attention mechanism; -Robust visual tracking with occlusion judgment and re-detection; -A scale adaptive object tracking algorithm with occlusion detection;- Double-channel object tracking with position deviation suppression] If the authors cannot employ these methods or compare their method with these methods, at least they could introduce/mention these novel technologies in related sections to improve the quality of the survey.

**Author response:** Thank you very much for your comments!

We carefully read the papers for your recommended. We have read the SiamCAm paper and the DCOT model. In terms of object tracking occlusion and background processing, we think that the processing and performance of these two algorithms are very good, and they are introduced and cited in our manuscript.

**Author action:** We have updated the manuscript based on your comments and the updates are as follows:

The field of multi-object tracking is mainly divided into two categories: offline tracking algorithms, such as MHT, Interacting Tracklets for Multi-Object Tracking, learning a neural solver for multiple object tracking[1-3], and online tracking algorithms, such as AS2RCF, DCOT, Tracktor++, CenterTrack, FairMot, and TransMOT[4-9].

Similar object interference in object tracking often leads to tracking drift, Kai Huang et al. [18] introduced a compensated attention model, which incorporates attention mechanisms in the feature extraction modules of both the template branch and search branch of the Siamese network. This model enhances the feature representation of both the target and the similar backgrounds simultaneously and improves the discriminative ability of the search branch towards the object.

[5]	Chu J, Tu X, Leng L, et al. Double-channel object tracking with position deviation suppression[J]. IEEE Access, 2019, 8: 856-866.

[18]	Huang K, Qin P, Tu X, et al. SiamCAM: A Real-Time Siamese Network for Object Tracking with Compensating Attention Mechanism[J]. Applied Sciences, 2022, 12(8): 3931.

**Reviewer#1, Concern # 5:** Please provide and label the reference indices of the compared methods in the figures and tables, such as Tables 1~6, and then the readers can judge whether the compared methods are SOTA.

**Author response:** Thank you very much for your comments! We have modified our manuscript as your comments.

**Author action:** Based on your comments, we have updated the reference citations for relevant algorithms as follows:

HOTA[24]: Geometric mean of detection accuracy and association accuracy. Averaged across localization thresholds.

The detection model of the MOT16 dataset was changed in this paper, and experiments were conducted using the DMP[26] detector and CenterNet[27] detector, and the experimental results are shown in Table 5 and Table 6.

Table 5: MOT16 Ablation Experiment DMP.

| Method | MOTA↑ | MOTP↓ | IDF1↑ | HOTA↑ | IDSW↓ | ML↓ | FN↓ | HZ |
| --- | --- | --- | --- | --- | --- | --- | --- | --- |
| Deepsort+DMP[19,26] | 28.4 | 78.4 | 34.2 | 27.8 | 458 | 318 | 85498 | **7** |
| Ours-Model | 28.8 | 78.4 | 35 | 28.3 | 395 | **312** | 84912 | **7** |
| Ours-Model+Ours-Association-strategy | **29.2** | **78.3** | **36.6** | **29.7** | **338** | **312** | **83601** | **7** |

Table 6: MOT16 Ablation Experiment CenterNet.

| Method | MOTA↑ | MOTP↓ | IDF1↑ | HOTA↑ | IDSW↓ | ML↓ | FN↓ | HZ |
| --- | --- | --- | --- | --- | --- | --- | --- | --- |
| Deepsort+CenterNet[19,27] | 54.6 | **80.4** | 60 | 47.7 | 594 | 90 | **52644** | **5** |
| Ours-Model | 58.1 | 81.6 | **62.3** | **49.4** | 488 | 91 | 53392 | **5** |
| Ours-Model+Ours-Association-strategy | **58.5** | 81.6 | 59 | 47.6 | **450** | **88** | 56116 | **5** |

[24]	Luiten J, Osep A, Dendorfer P, et al. Hota: A higher order metric for evaluating multi-object tracking[J]. International journal of computer vision, 2021, 129: 548-578.

[26]	Sadeghi M A, Forsyth D. 30hz object detection with dpm v5[C]//Computer Vision–ECCV 2014: 13th European Conference, Zurich, Switzerland, September 6-12, 2014, Proceedings, Part I 13. Springer International Publishing, 2014: 65-79.

[27]	Duan K, Bai S, Xie L, et al. Centernet: Keypoint triplets for object detection[C]//Proceedings of the IEEE/CVF international conference on computer vision. 2019: 6569-6578.

**Reviewer#1, Concern # 6:** Why are the compared methods different in the tables and figures? E.g. Tables 7 and 8. All the comparisons should be fair, objective and comprehensive rather than biased, subjective and selective.

**Author response:** Thank you extremely much for your valuable comments!

We checked and added all comparison methods to ensure an unbiased comparison. There are more algorithms in the MOT17 dataset than MOT16. Most of these algorithms are new algorithms in recent years, and some of them have not been tested on the MOT16 dataset. In future, we will improve our manuscript. We updated our comparison algorithm as your comments.

**Author action:** The experimental comparison is updated as follows:

Table 7: MOT16 test set comparison experiment.

| Method | MOTA↑ | IDF1↑ | HOTA↑ | IDSW↓ | ML↓ | FN↓ |
| --- | --- | --- | --- | --- | --- | --- |
| OUTrack_fm_p[28] | **69.3** | **66.7** | **54.5** | 1298 | **124** | **41177** |
| Lif_TsimInt[29] | 57.5 | 64.1 | 49.6 | **335** | 263 | 72868 |
| MPTC[32] | 62.9 | 65.1 | 51.1 | 685 | 240 | 63565 |
| Tracktor++[6] | 54.4 | 52.5 | 42.3 | 682 | 280 | 79149 |
| KCF16[30] | 48.8 | 47.2 | 37.2 | 906 | 289 | 86567 |
| Ours | 64.0 | 62.4 | 51.0 | 838 | **124** | 47849 |

Table 8: MOT17 test set comparison experiment.

| Method | MOTA↑ | IDF1↑ | HOTA↑ | IDSW↓ | ML↓ | FN↓ |
| --- | --- | --- | --- | --- | --- | --- |
| PermaTrack[31] | **73.1** | 67.2 | 54.2 | 3571 | 450 | **123508** |
| OUTrack_fm_p[26] | 69.0 | 66.8 | 55.5 | 3639 | 615 | 140457 |
| Byte_Track[15] | 67.4 | **70** | **56.1** | 1331 | 735 | 172636 |
| MPTC[32] | 62.6 | 65.8 | 51.7 | 4074 | 750 | 198338 |
| Lif_TsimInt[29] | 58.2 | 65.2 | 50.7 | **1022** | 791 | 217944 |
| TADN[33] | 54.6 | 49.0 | 54.8 | 4472 | 464 | 141580 |
| Tracktor++[6] | 53.5 | 52.3 | 42.1 | 2072 | 861 | 248047 |
| JBNOT[34] | 52.6 | 50.8 | 41.3 | 3050 | 844 | 232659 |
| FAMNet[35] | 52.0 | 48.7 | 0.0 | 3072 | 787 | 253616 |
| Ours | 63.2 | 61.8 | 50.5 | 2616 | **414** | 157581 |

**Reviewer#2, Concern # 1:** English writing needs further improvement. The structure of this paper needs to be optimized and the METHOD section (current sections 2 and 3) should be highlighted. At present, it is difficult for readers to find the author's scientific discovery or technological innovation.

**Author response:** Thank you for your suggestion!

We appreciate your feedback. In response to reviewers and readers, we have made several revisions to the manuscript to better showcase our innovations and techniques. Firstly, we have added new titles and modified the titles of existing methods to accurately reflect the advancements we have made.

In the second part, we have included a subtitle called "Fusion of cyclic shift and self-attention mechanism" to highlight our efforts in optimizing the model. This section elaborates on our approach and the benefits of incorporating these two techniques.

Furthermore, we have thoroughly revised the main content and subtitles of Section III to present our newly proposed target association strategy. Additionally, we have introduced the use of the least squares method to address the occlusion problem, further emphasizing our novel ideas.

We believe that these revisions will effectively communicate our work and innovations to both reviewers and readers. Thank you again for your valuable input!

**Author action:** Based on your review comments, our revisions are as follows:

**2.1. Fusion of cyclic shift and self-attention mechanism**

**3. High value prediction box target association strategy**

We propose a more robust association strategy, which selects an appropriate trajectory prediction algorithm based on the number of lost frames caused by the occlusion of the target detection box. The high-value prediction boxes are used for association, effectively reducing the number of identity switches during the tracking process.

**3.1. High value prediction box selection and matching**

**3.2. Introducing the least square method to reduce ID switching under frequent occlusion**

**Reviewer#2, Concern # 2:** In Fig. 1, “Cycle-Attention” module has only one input, but in Fig. 2, two inputs appear. Additionally, it is better to draw the output arrow in Fig. 2. In the figures or text, there is no description of the internal structure of the “Self-Attention” module in Fig. 1.

**Author response:** Thank you very much for your comments!

We updated Figure 1 and Figure 2 in the revised manuscript. We refined the module part in Figure 1，then modified the input for Figure 1 and Figure 2. And the relevant structure diagram of self-attention is given in Figure 2.

**Author action:** We have updated the model diagram in our revised manuscript, and the updated model diagram is shown as:

Fig.1:Feature extraction model diagram.

Fig.2: Cycle-attention module.

**Reviewer#2, Concern # 3:** Please indicate which attention mechanism or its internal structure is used for the blue attention module on the right in Fig. 2.

**Author response:** Thank you extremely much for your valuable comments!

In our revised manuscript, we used the self-attention mechanism. In Figure 2, we have redrawn its internal structure and marked the type of attention what we used.

**Author action:** Based on your comments, we have modified the model diagram as follows:

Fig.2: Cycle-attention module.

**Reviewer#2, Concern # 4:** How is the “cycle shift” operation done in Fig. 2 and what is its purpose? How to construct a large number of negative samples through cycle shift in the “Cyclic-Attention” module? It's better to introduce it. Will generating these negative samples and their subsequent processing introduce a lot of computational overhead? Has the author proposed methods to improve computational efficiency?

**Author response:** Thank you for your valuable comments!

We have added new modules in Figure 2 to explain our cyclic shift steps, as well as the display diagram of positive and negative samples formed after cyclic shift, and added related text descriptions. During the experiment, we designed this module in order to improve the robustness and accuracy of the model. Since the module constructs negative samples for each tracking target and extracts global feature information through the attention mechanism, the accuracy is improved. At the same time, it brings computational overhead, but the computational overhead is within an acceptable range, and we will improve this problem in future work.

**Author action:** Based on your comments, we have modified the model diagram as follows(Figure 2) and added a method for cyclic shifting, these are:

Finally, ResNet50 is used as the backbone for training, and the Cycle-Attention module is shown in Figure 2. In this paper, the two-dimensional image is reduced in dimension, and the number of cyclic shifts is 1/16, 1/8, and 1/4 of the original image.

Fig.2: Cycle-attention module.

**Reviewer#2, Concern # 5:** Figs. 1 and 2 should provide legends for ○+ and ○× operations. What does the ○× symbol in Fig. 6 mean? What does “confirm d” in Fig. 6 mean?

**Author response:** Thank you very much for your valuable comments!

We have revised the flowchart in Fig.6 based on your comments in the revised manuscript.

**Author action:** We have modified the flow chart, and the modified part is shown in the Figure 6, that is

Fig.6. Matching flow chart.

**Reviewer#2, Concern # 6:** Please unify the use of the term “equation”. In Equations 10 and 11, “formula” is used, but all other equations use “equation”.

**Author response:** Thank you very much for your valuable comments!

Based on your suggestions, we have carefully checked and revised our manuscript.

**Author action:** We have modified the use of equation in the revised manuscript.

**Reviewer#2, Concern # 7:** More in-deep analyses, discussion and comparison are needed in Experiments. Competitive results with recent works are needed.

**Author response:** Thank you very much for your valuable comments!

We have added section 4.3 to analyze and discuss the model and association strategy we designed, analyze the reasons why our algorithm achieves better performance on the dataset, and analyze the reasons why some indicators of the algorithm decline on the MOT15 dataset. In the 4.1 Ablation experiment, we added Table 4 and added the ablation experiment of the algorithm in this paper on the MOT17 data set. Added a more detailed HOTA indicator comparison chart.In the 4.2 comparison experiment, we added the published SOTA model to the MOT16 and MOT17 datasets to compare the number of identity switching indicators, checked the comparison algorithms on the two datasets, and added the same Experimental results of the algorithm.

**Author action:** We have updated our revised manuscript as your comments, and these are:

Experimental results are shown in Table 3, Table 4 and Table 5, where the detection model confidence threshold is set to 0.3. The MOT17 dataset uses the default DPM[26], Faster-RCNN[27] and SDP[28] three detection inputs.

Table 4: MOT17 Ablation Experiment.

| Method | MOTA↑ | MOTP↓ | IDF1↑ | HOTA↑ | IDSW↓ | ML↓ | FN↓ | HZ |
| --- | --- | --- | --- | --- | --- | --- | --- | --- |
| Deepsort[19] | 72.1 | 81.3 | 69 | 56.8 | 2299 | 185 | 124741 | **6** |
| Ours-Model | 72.4 | 81.3 | **70** | **58** | 1961 | **177** | **120243** | 4 |
| Ours-Model+Ours-Association-strategy | **72.5** | **81.2** | 69.5 | 57.1 | **1841** | **177** | 124311 | 4 |

On the MOT17 dataset, the total number of identity switches is reduced by 20%. The HOTA index is evaluated through three sub-tasks detection, association and positioning. In Fig 13, the algorithm in this paper is compared with the DeepSORT algorithm on the HOTA index. In terms of association accuracy AssA and HOTA score, for different positioning the similarity threshold alpha algorithm in this paper has been improved.

**Fig 13. Comparison results of HOTA indicators.**

Table 8: MOT16 test set comparison experiment.

| Method | MOTA↑ | IDF1↑ | HOTA↑ | IDSW↓ | ML↓ | FN↓ |
| --- | --- | --- | --- | --- | --- | --- |
| OUTrack_fm_p[30] | **69.3** | **66.7** | **54.5** | 1298 | **124** | **41177** |
| Lif_TsimInt[31] | 57.5 | 64.1 | 49.6 | **335** | 263 | 72868 |
| MPTC[34] | 62.9 | 65.1 | 51.1 | 685 | 240 | 63565 |
| Tracktor++[6] | 54.4 | 52.5 | 42.3 | 682 | 280 | 79149 |
| KCF16[32] | 48.8 | 47.2 | 37.2 | 906 | 289 | 86567 |
| Ours | 64.0 | 62.4 | 51.0 | 838 | **124** | 47849 |

Table 9: MOT17 test set comparison experiment.

| Method | MOTA↑ | IDF1↑ | HOTA↑ | IDSW↓ | ML↓ | FN↓ |
| --- | --- | --- | --- | --- | --- | --- |
| PermaTrack[33] | **73.1** | 67.2 | 54.2 | 3571 | 450 | **123508** |
| OUTrack_fm_p[26] | 69.0 | 66.8 | 55.5 | 3639 | 615 | 140457 |
| Byte_Track[15] | 67.4 | **70** | **56.1** | 1331 | 735 | 172636 |
| MPTC[34] | 62.6 | 65.8 | 51.7 | 4074 | 750 | 198338 |
| Lif_TsimInt[31] | 58.2 | 65.2 | 50.7 | **1022** | 791 | 217944 |
| TADN[35] | 54.6 | 49.0 | 54.8 | 4472 | 464 | 141580 |
| Tracktor++[6] | 53.5 | 52.3 | 42.1 | 2072 | 861 | 248047 |
| JBNOT[36] | 52.6 | 50.8 | 41.3 | 3050 | 844 | 232659 |
| FAMNet[37] | 52.0 | 48.7 | 0.0 | 3072 | 787 | 253616 |
| Ours | 63.2 | 61.8 | 50.5 | 2616 | **414** | 157581 |

**4.3. Results evaluation**

When tracking algorithms such as SORT and DeepSORT use Kalman filters to predict trajectories, frequent occlusions can lead to ID switches in the tracked targets. In this paper, the least squares method and Kalman filter are introduced to handle long-term and short-term occlusions separately, resulting in more reasonable predictions of object tracking bounding boxes.

The average density of the object tracking dataset is relatively high, and frequent occlusions lead to more background clutter. The feature information extracted by the ReID model is often insufficient. This paper designs a dual-path self-attention mechanism module that combines cyclic shift operations with self-attention mechanisms. This module exhibits higher robustness and feature extraction capabilities. In the ablation experiment (Section 4.1), the feature extraction model proposed in this paper shows improvements in accuracy metrics. The more accurate the object detection model, the higher the accuracy improvement achieved by the feature extraction model in this paper.

Common object tracking algorithms usually consider cases where the number of object detection frames is low (during frequent occlusions) or when the tracking boxes have been absent for a long time. In the case of a low number of detection frames, the detection box is not used, while in the case of a long absence of tracking boxes, the detection boxes are often neglected to prevent computational overhead, resulting in increased identity switches of the tracked targets. The correlation strategy designed in this paper considers these types of detection boxes and effectively reduces the number of identity switches during object tracking. As shown in Section 3.3, the high-value detection boxes selected in this paper are used only once during target association, reducing identity switches while preventing false detections and additional costs. In the ablation and comparative experiments, the algorithm proposed in this paper achieves a lower ML while reducing the number of identity switches.

MOT15: MOT15 consists of 11 sequences in various indoor or outdoor public pedestrian scenes. Both the model and correlation strategy in this paper reduce the number of identity switches. This dataset has a lower resolution, resulting in less pixel information and the loss of some details and crucial information. The model proposed in this paper uses cyclic shifts to construct negative samples, which are suitable for low-resolution targets but are more susceptible to background noise, leading to decreased accuracy. In future work, super-resolution reconstruction or image enhancement methods will be considered to make the model applicable to low-resolution datasets.

MOT17: The MOT17 dataset contains seven different sequences in both the training and testing sets. It has a higher average target density, and frequent occlusions occur during target motion. In the ablation experiment (Section 4.1), the proposed model and correlation strategy in this paper reduce the number of identity switches. In the comparative experiment (Section 4.2), the algorithm proposed in this paper achieves higher accuracy and fewer identity switches compared to recent algorithms.

Figures 7, 8, 9, and 12 demonstrate the tracking results of the correlation strategy in this paper when dealing with scenarios of fewer detection frames and more disappearing frames. Additionally, we present selected tracking results for occluded targets, such as in Figure 14 and Figure 15. The proposed algorithm in this paper can correctly assign the correct IDs even in cases of complete occlusion (ID 36) and partial occlusion (ID 31,38), effectively reducing the number of switches during object tracking.

Fig.14: The result of the target complete occlusion experiment.

Fig.15: Partial occlusion experiment results of the target.

**Reviewer#2, Concern # 8:** Overall, the literature review should be improved. The authors need to review and discuss visual object tracking (For example, DOI: 10.1016/j.asoc.2022.108485; 10.1016/j.compeleceng.2022.107730; 10.1007/s12652-020-02572-0; 10.1109/LSP.2023.3238277 ) and detection (DOI: 10.22967/HCIS.2022.12.023; 10.3233/AIS-220038; 10.1007/s11554-022-01252-w) algorithms. It is suggested that the related papers should be cited.

**Author response:** Thank you very much for your valuable comments!

We carefully read all papers as your recommended. We read the paper that title is ”An object tracking framework with recapture based on correlation filters and Siamese networks”, and we found that the algorithm is valuable for occlusion problem and background clutter problem, and its performance on related dataset can reach SOTA. It is suitable for our further study. So we added them in our revised manuscript.

**Author action:** We have updated our revised manuscript as your comments, and these are:

The field of multi-object tracking is mainly divided into two categories: offline tracking algorithms, such as MHT, Interacting Tracklets for Multi-Object Tracking, learning a neural solver for multiple object tracking[1-3], and online tracking algorithms, such as AS2RCF, DCOT,Tracktor++, CenterTrack, FairMot, and TransMOT[4-9].

Zhang Jianming et al. [17] combined deep features with handcrafted features and proposed a new Robustness Criterion for evaluation. They used an adaptive threshold to determine whether to use the Siamese network for re-detection, effectively addressing occlusion and background clutter issues in object tracking and achieving state-of-the-art performance.

[4]	Zhang J, He Y, Wang S. Learning Adaptive Sparse Spatially-Regularized Correlation Filters for Visual Tracking[J]. IEEE Signal Processing Letters, 2023.

[17]	Zhang J, Sun J, Wang J, et al. An object tracking framework with recapture based on correlation filters and Siamese networks[J]. Computers & Electrical Engineering, 2022, 98: 107730.
